# Release and Degradation of Microencapsulated Spinosad and Emamectin Benzoate

**DOI:** 10.1038/s41598-017-11419-2

**Published:** 2017-09-07

**Authors:** Bin Bin Huang, Shao Fei Zhang, Peng Hao Chen, Gang Wu

**Affiliations:** Key Laboratory of Biopesticide and Chemical Biology (Ministry of Education), Fujian Agriculture and Forestry University, Fuzhou, 350002 China

## Abstract

The dynamics of release and degradation of the microencapsulation formulation containing spinosad (SP) and emamectin benzoate (EM) were evaluated in the present study. SP and EM were microencapsulated using biodegradable poly-lactic acid (PLA) as the wall material. Their release from and degradation within the prepared SP and EM microspheres (SP-EM-microspheres) were studied. It was found that the encapsulation significantly prolonged the insecticide release. The release could be further extended if the external aqueous phase was pre-saturated with the insecticides and the microspheres were additionally coated with gelatin. On the other hand, increasing the water content of the emulsion or the hydrophilic polycaprolactone (PCL) content in the PLA/PCL mixture accelerated the release. Due to the photolysis and hydrolysis of SP and EM by sunlight, the toxicity of the non-encapsulated insecticides in water declined continuously from 0 through the 9^th^ day (d), and dissipated in 13 d. In contrast, an aqueous suspension containing 5% SP-EM-microspheres maintained a mostly constant toxicity to *Plutella xylostella* for 17 d. The biodegradable SP-EM-microspheres showed significantly higher long-term toxicity to *P. xylostella* due to lower release, reduced photolysis and hydrolysis of the encapsulated insecticides, which were affected by the varied preparation conditions.

## Introduction

Insects can become more resistant to insecticides applied in a large quantity for a prolonged period of time^1^. Cocktailed applications with a combination of different insecticides may improve their efficacy. Commercially, such formulations can indeed frequently delay the development of pest’s resistance to the insecticides, and at the same time, enhance their toxicity against the insects.

Both spinosad (SP) and emamectin benzoate (EM) were biological insecticides highly effective against a wide range of insects. SP was a kind of macrolide compounds extracted from the fermentation broth of *Saccharopolyspora spinosa*. EM was the synthesis of derivatives by abamectin which was isolated from the fermentation broth of *Streptomyces avermitilis* MA-4680. The targeted pest spectrum included Lepidopteran, Diptera and Thysanopteran insects for SP, and of Coleopteran, Dipteran, Homopteran and Lepidopteran insects for EM. The recommended dosages in the fields are 300–600 ml/ha for 60 g/L SP suspension concentrate, and 150–225 g/ha for 5.7% EM water dispersible granules. Unfortunately, various pests have reportedly developed significant resistance to SP around the world^2–5^. To control pests in the field, SP has to be formulated at high concentration and in combination with other active components. For instance, 48%, rather than the conventional 2.5%, of SP suspension concentrate (SC) is used in China. Meanwhile, the efficacy of EM against the target pests remains highly active at concentrations ranging from as low as 0.5% to 2.5%, even with the heavy application for more than a decade in China^6^. The insecticide-resistance of the target pests was effectively suppressed by combining SP and EM, while the insecticide’s efficacy significantly enhanced^7^.

On the other hand, both SP and EM are sensitive to sunlight. They degrade promptly when exposed to the sun^8, 9^. Hirech^10^ suggested that this shortcoming could be partially remedied by microencapsulation, which is a technology that separates a core material from the external mass by forming a “microsphere” or “microcapsule”. A microsphere is when a solid core material is enclosed in a polymeric network, while a microcapsule is a liquid/or solid core surrounded by a polymeric wall^11, 12^. Besides, the latter is formed using a non-biodegradable material, such as polyurea^13^ or polyurethane^14^ as the wall material, as widely found in formulating controlled-release insecticides around the world^15–20^. Being non-biodegradable, these materials cause serious environmental concerns about their long-term, heavy applications. It would, therefore, be desirable to use natural, biodegradable high polymers^21–24^ or chemical synthetic polyesters that are biodegradable for the purpose^25–33^.

Liu *et al*.^34^ suggested that the rates of release and degradation of SP and EM within SP and EM microspheres (SP-EM-microspheres) were reduced due to the coating. In the past, most attempts applied a single insecticide to the core, as the preparation of microspheres with multiple ingredients was rather complicated. Our previous studies used biodegradable poly-lactic acid (PLA) for the wall material to prepare microspheres containing either SP^28, 29^ or EM^35^. However, the physicochemical characteristics of the SP-EM-microspheres, such as the insecticide loading, entrapment rate and mean diameter, morphology, insecticide release, degradation and toxicity, must be well understood before the field applications.

In this paper, the effects of preparation conditions on the physicochemical characteristics, insecticide release and degradation of the SP-EM-microspheres are presented. Also, both short- and long-term toxicities of the microspheres on *Plutella xylostella*, one of the most devastating pests in the vegetable fields worldwide, were evaluated under sunlight exposure.

## Results

### Comparison of methodologies

The release of the SP and EM into the microspheres increased with time, from the second to twenty fourth day (d) (Duncan’s tests, *P* ≤ 0.05) (Table 1). No significant differences between SP and EM in 2–7 d. But, from the 10^th^ to the 24^th^ d, the release rate of EM was significantly higher than that of SP (*t*-tests, *P* ≤ 0.05). There were no significant differences in the rates determined by either high-performance liquid chromatography (HPLC) or spectrophotometer (*t*-tests, *P* ≤ 0.05). The results also indicated that spectrophotometer could be used to determine the release rate of the two insecticides in the release medium (Table 1).

**Table 1:** Release rate by different determination methods.

Release time (d)	Release rate (%) [high performance liquid chromatography (HPLC) method]			Cumulative release rate of SP + EM (%) (spectrophotometer method)	Deviation of spectrophotometer method from HPLC method (%)
	spinosad (SP)	emamectin benzoate (EM)	SP + EM		
2	34.7 ± 1.24hA	35.5 ± 1.55iA	34.9 ± 1.26iA	35.2 ± 1.02 g	0.849
4	49.4 ± 0.87gA	48.2 ± 0.88hA	49.2 ± 0.87hA	48.0 ± 2.40fA	−2.304
7	53.6 ± 1.58fA	54.7 ± 0.50gA	53.8 ± 1.30gA	54.2 ± 0.37eA	0.743
10	57.9 ± 0.72eA	62.1 ± 0.26fB	58.8 ± 0.53fA	61.5 ± 1.70dA	4.636
13	60.3 ± 0.93dA	65.8 ± 0.88eB	61.4 ± 0.91eA	63.2 ± 2.00cdA	2.906
15	61.7 ± 1.38cdA	69.0 ± 0.69 dB	63.2 ± 1.03dA	65.7 ± 1.27bcA	3.968
18	63.4 ± 0.75cA	73.5 ± 1.28cB	65.4 ± 0.79cA	68.1 ± 2.96bA	4.075
20	68.1 ± 0.74bA	79.8 ± 0.26bB	70.4 ± 0.57bA	72.9 ± 1.86 aA	3.520
24	71.3 ± 0.77 aA	83.3 ± 1.21aB	73.7 ± 0.38 aA	76.1 ± 2.49 aA	3.281

Note: Deviation = [(Release rate by spectrophotometer method - Release rate by HPLC method)/Release rate by HPLC method] × 100. Different lower-case letters (such as a, b, c, d, e, f, g, h, and i) in a same column indicated significant differences among release rates at different release times (Duncan’s tests, *P* ≤ 0.05). Different capital letters (such as A and B) indicated significant differences on release rate between SP and EM by using HPLC, or those of SP + EM between HPLC and spectrophotometer methods (*t*-test, *P* ≤ 0.05).

### Insecticide release affected by the condition of external aqueous phase

**Figure 1:**
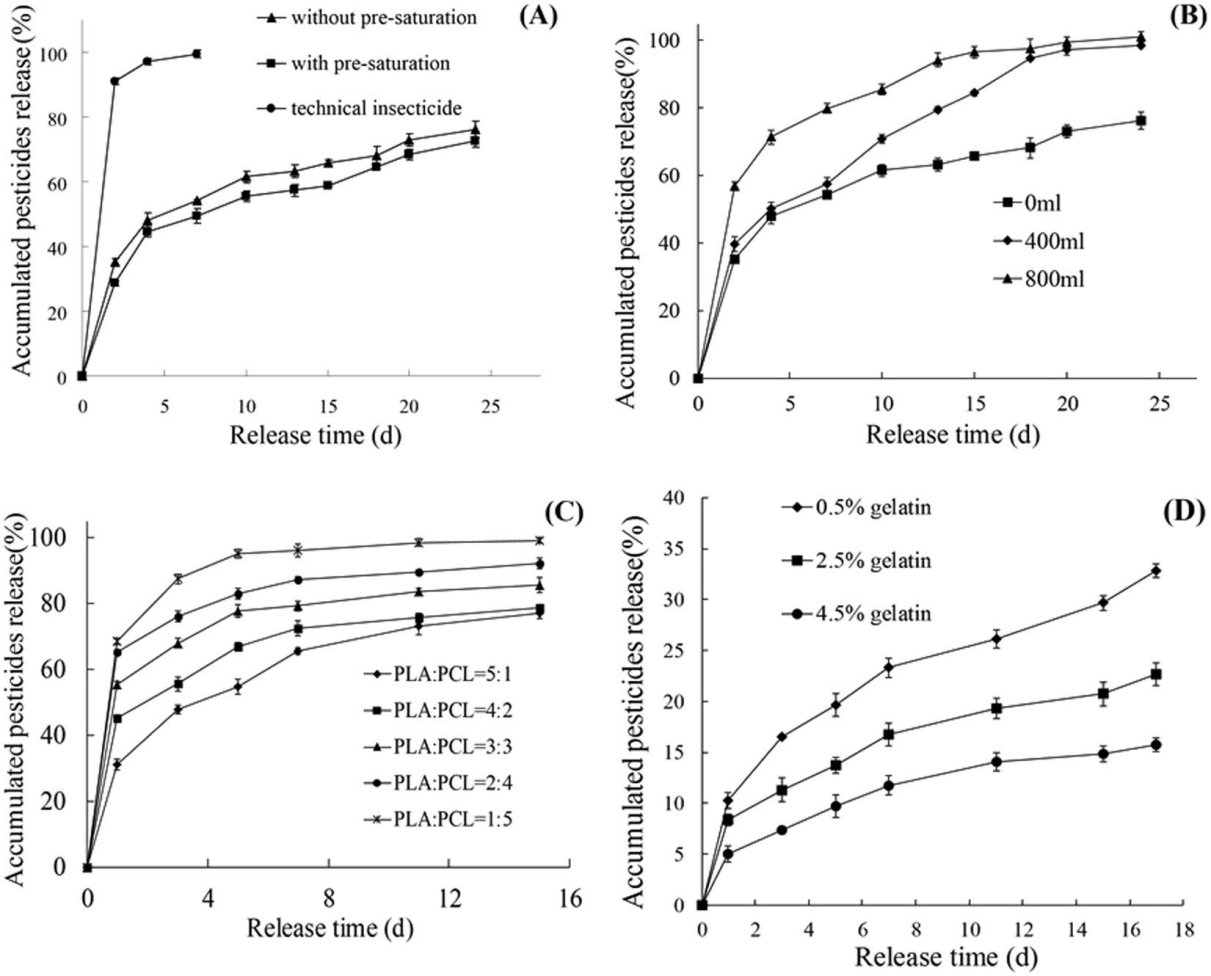
Release rates of SP + EM in SP-EM-microspheres prepared under different conditions. SP + EM contents were determined by spectrophotometer method. In typical solvent evaporation method^31^, the external aqueous phase was not saturated with SP and EM, the emulsion was not added with additional water, PLA was the lone wall material, and no gelatin was applied for coating the microspheres. (**A**) External aqueous phase with or without insecticides saturation. (**B**) With or without added distilled water in emulsion. (**C**) PLA and PCL in different ratios for microsphere wall. (**D**) Concentrations of gelatin for microsphere coating. In (**A**), (**B**) and (**D**), only PLA was used. In (**A**), (**C**) and (**D**), no water was added to emulsion.

The release of SP and EM from the SP-EM-microspheres was significantly lowered as compared to the non-microencapsulated samples (Fig. 1A). Whether or not the external aqueous phase was saturated with the insecticides did not show significant variations in the insecticide loading, entrapment rate, mean diameter, and span (Table 2) or morphology of the microspheres (Fig. S1). On the other hand, the SP-EM-microspheres suspension prepared with a saturated external aqueous phase displayed significantly lower insecticide release and significantly higher *T*_50_, which was the time when 50% release rate was reached, otherwise, in 2–25 d (Table 2, Fig. 1A). The results indicated that the SP-EM-microspheres retarded the insecticide release, and that, by saturating the external aqueous phase with the insecticides, the efficacy of the insecticides could be enhanced.

**Table 2:** Insecticide release of spinosad (SP) and emamectin benzoate (EM)-micropheres (SP-EM-microspheres) affected by preparation conditions.

Conditions	Entrapment rate of SP + EM (%)^(a)^	Loading of SP + EM (%)^(a)^	Mean diameter of microspheres (μm)	Span	Kinetic equation of release curve of SP + EM^(b)^	*T*_50_ (d)^(b)^
**A: external aqueous phase**						
Not saturated with SP + EM	84.5 ± 0.67a	40.6 ± 0.32a	7.19 ± 0.27a	1.13 ± 0.023a	M_t_/M_z_ = 30.5t^0.29^	5.45
Saturated with SP + EM	87.9 ± 0.78a	42.2 ± 0.38a	7.11 ± 0.21a	1.11 ± 0.016a	M_t_/M_z_ = 23.6t^0.35^	8.86
**B: distilled water addition (ml)**						
0	84.5 ± 0.67b	40.6 ± 0.32b	7.19 ± 0.27b	1.13 ± 0.023a	M_t_/M_z_ = 30.5t^0.29^	5.45
400	88.3 ± 0.92a	41.8 ± 0.44a	7.58 ± 0.18ab	1.24 ± 0.082a	M_t_/M_z_ = 30.4t^0.38^	3.70
800	89.6 ± 0.73a	42.4 ± 0.35a	7.71 ± 0.22a	1.24 ± 0.037a	M_t_/M_z_ = 50.5t^0.23^	0.961
**C: PLA / PCL ratio**						
5:1	82.6 ± 0.95ab	39.7 ± 0.45ab	7.16 ± 0.26a	1.14 ± 0.012a	M_t_/M_z_ = 33.7t^0.32^	3.45
4:2	83.4 ± 0.74b	40.1 ± 0.36b	7.19 ± 0.27a	1.20 ± 0.061ab	M_t_/M_z_ = 46.1t^0.20^	1.47
3:3	81.4 ± 0.81a	39.1 ± 0.39a	7.35 ± 0.23a	1.16 ± 0.013ab	M_t_/M_z_ = 55.9t^0.17^	0.51
2:4	82.2 ± 0.79ab	39.5 ± 0.38ab	7.05 ± 0.19a	1.18 ± 0.027b	M_t_/M_z_ = 65.8t^0.13^	0.14
1:5	81.1 ± 0.70a	39.0 ± 0.34a	6.97 ± 0.31a	1.17 ± 0.042ab	M_t_/M_z_ = 72.3t^0.13^	0.060
**D: gelatin concentration (%)**						
0	84.5 ± 0.67c	40.6 ± 0.32c	7.19 ± 0.27a	1.13 ± 0.023a	M_t_/M_z_ = 30.5t^0.29^	5.45
0.5	80.7 ± 1.29b	38.8 ± 0.62b	7.26 ± 0.31a	1.14 ± 0.049ab	M_t_/M_z_ = 10.3t^0.40^	53.7
2.5	75.8 ± 0.95a	36.4 ± 0.42a	7.45 ± 0.27a	1.21 ± 0.025b	M_t_/M_z_ = 7.60t^0.39^	129
4.5	74.2 ± 0.81a	35.7 ± 0.39a	7.65 ± 0.26a	1.18 ± 0.015b	M_t_/M_z_ = 4.51t^0.45^	212

Note: (a): Contents of SP + EM determined by HPLC. (b): Contents of SP + EM determined by spectrophotometer. “M_t_/M_z_” and “t” represent cumulative quantity of insecticides release (%) and release time (d), respectively. *T*_50_ represents the time (d) when cumulative insecticides release reached 50%. Kinetic equations of insecticides release for condition A to D and *T*_50_ were calculated based on data in Fig. 1A to D, respectively. Correlation coefficients for two fitted equations were higher than 0.99. Different letters (such as a, b, and c) in a same column represents significant difference (*t*-test, *P* ≤ 0.05).

### Insecticide release affected by water addition in the emulsion

Although the insecticide loading, entrapment rate and mean diameter of the SP-EM-microspheres increased appreciably (*P* ≤ 0.5) when distilled water was added to the emulsion, they were not significant in general (Table 2). Nor did the addition of 400 ml distilled water result in significant changes in the microsphere’s morphology (Fig. S1). However, the water addition significantly increased the insecticides release of the SP-EM-microspheres during the 10 to 25 d. When 800 ml distilled water were added, the release increased from the second to twenty fifth days (Fig. 1B). On the other hand, *T*_50_ decreased with the water added during the same period.

### Insecticide release affected by PLA/PCL ratio

When polycaprolactone (PCL) was used together with PLA as the wall materials, the insecticide release increased, while *T*_50_ decreased, with increased PCL (Table 2, Fig. 1C). On the other hand, no significant change was observed on the insecticide loading, entrapment rate, mean diameter and span or on the morphology of the microspheres when PLA:PCL was 5:1 (Fig. S1). It suggested that the insecticide release could be accelerated by incorporating PCL, a polymer less hydrophobic than PLA, in preparing the microspheres.

### Insecticide release affected by gelatin coating

To reduce the initial bursts of insecticide release from the microspheres, the SP-EM-microspheres were dipped in solutions containing varying amounts of gelatin to form a protective coating. The coating induced no significant variation in the morphology when the gelatin concentration was 2.5% (Fig. S1). However, it significantly eliminated the initial bursts of the insecticide release and provided a stable, continuous flow of SP and EM afterward. The concentration of gelatin affected the insecticide release in different manners. At the 18 d, 0.5%, 2.5%, and 4.5% gelatin solutions yielded microspheres with a release rate of 31.9%, 22.7%, and 15.7%, respectively (Fig. 1D), and *T*_25_ [the time (d) when cumulative insecticides release reached 25%] of 9.3 d, 21.7 d, and 45.3 d, respectively (not presented in Table 2). The *T*_50_ of gelatin-coated SP-EM-microspheres (i.e., 53.7) was far greater than that of the control (5.45) (Table 2, Fig. 1D and A). It appeared that high concentration of gelatin would lower the insecticide release from the SP-EM-microspheres.

### Photolysis stability

**Figure 2:**
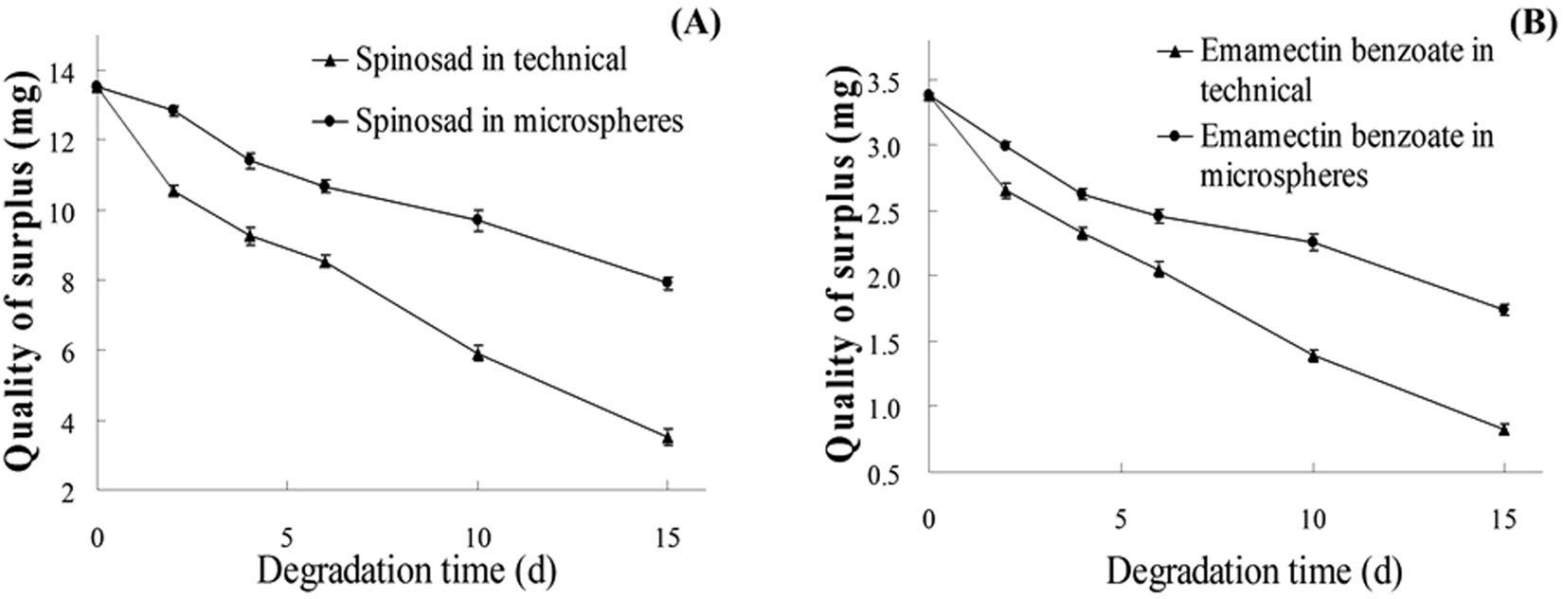
Light degradation of insecticides in SP-EM-microspheres or as naked insecticides.

Upon exposure to natural light, the insecticides in the SP-EM-microspheres degraded significantly less than their non-encapsulated counterparts mixed with blank microspheres. In 15 d, the naked SP and EM degraded 73.9% and 75.5%, respectively; whereas, the insecticides enclosed in the microspheres degraded 41.4% and 48.5%, respectively (Fig. 2). The half-life of the naked SP and EM was 7.92 d, and 7.26 d, respectively; but, protected by the encapsulation it became 19.88 d, and 16.16 d, respectively (Table 3). Consequently, the microencapsulation rendered a significant advantage on preserving the insecticide efficacy by reducing the effect of sun photolysis.

**Table 3:** Kinetic equation of light degradation of insecticides. Note: Contents of SP + EM determined by HPLC.

Insecticide	Formulation	Kinetic equation	Correlation coefficient	Half-life (d)
SP	Non-encapsulated	Q_t_ = 13.5e^−0.088t^	0.99	7.92
	In SP-EM-microspheres	Q_t_ = 13.5 e^−0.036t^	0.99	19.9
EM	Non-encapsulated	Q_t_ = 3.36 e^−0.096t^	0.99	7.26
	In SP-EM-microspheres	Q_t_ = 3.37 e^−0.046t^	0.98	16.2

Kinetic equations based on degradation curve shown in Fig. 2.

### Toxicity bioassay

Based on the mortality at 48 h, the toxicity regression equations and lethal concentration 50 (LC_50_) were calculated. The LC_50_ (95% confidence limit) of mixture of 2.5% SP suspension concentrate and 1.5% EM emulsifiable concentrate (MX) (maintaining an SP:EM = 4:1) and that of SP-EM-microspheres suspension (MS) was 0.43 (0.17–1.15), and 0.94 (0.43–2.03) mg/L, respectively. The two preparations were kept under sunlight for different durations. The photolysis and hydrolysis of SP and EM were examined. At 0 d, i.e., immediately after the preparations were made, there was no photolysis and hydrolysis. At that time, the toxicity of MS was 60.2% of that of MX, even though the initial insecticide concentrations of the two were same at 1.2 mg/L. However, after 9 d, the toxicity of MX declined significantly and had practically disappeared on the final day of the test. In contrast, MS maintained a similar toxicity for 17 d (Table 4).

**Table 4:** Photolysis and hydrolysis of SP and EM in aqueous preparations under sunlight.

Initial concentration of SP + EM (mg/L)	Degradation time under sunlight (d)	Mortality (%)(48 h)	
		MX (SP:EM = 4:1)	SP-EM-MS
1.2	0	86.7 ± 5.77 aA	52.2 ± 5.08aB
1.2	2	63.3 ± 8.79b	—
1.2	7	27.8 ± 8.40c	—
1.2	9	11.1 ± 3.81dA	50.0 ± 3.30aB
1.2	13	0.0eA	53.3 ± 3.35aB
1.2	17	—	55.6 ± 10.2a
1.2	21	—	41.1 ± 10.2b

Note: MX = the mixture of 2.5% SP suspension concentrate and 1.5% EM emulsifiable concentrate. Initial insecticide concentrations of MS and MX were same at 1.2 mg/L. Different lower-case letters (such as a, b, c, d, and e) indicate significantly different pest mortalities between insecticide preparations of different degrees of degradation due to varied durations of sun exposure (Duncan’s tests, *P* ≤ 0.05). Different capital letters (such as A and B) indicate significantly different pest mortalities between two preparations that underwent a same duration of sun exposure (*t*-test, *P* ≤ 0.05).

## Discussion

In our previous research, PLA was confirmed to be an excellent wall material to prepare SP-EM-microspheres as compared to other kinds of wall material, and the preliminary optimized preparation condition for SP-EM-microspheres was established^34–36^. Two conditions, i.e., external aqueous phase (with or without saturated SP + EM) and the volume of distilled water addition, was confirmed to be essential for preparing the SP-EM-microspheres. Therefore, the mean diameter, entrapment, loading and span as affected by the two conditions were studied. According to our observation, not so great effects of PLA/PCL ratio and gelatin concentration on the insecticide loading, entrapment rate, mean diameter and span were found. However, the release and degradation process of SP-EM-microspheres were focused on in the present study.

### External aqueous phase pre-saturated with insecticide might enable uniform distribution of insecticide within the microsphere

Spenlehauer *et al*.^37^ found that the drug entrapment rate was markedly improved in their biodegradable cisplatin-poly (d, l-lactide) microspheres when the aqueous phase was saturated with cisplatin in the process of using a solvent evaporation method in particular. Our study also found that the insecticide loading and the entrapment rate of the SP-EM-microspheres increased slightly, while the insecticides release declined significantly when the external aqueous phase was pre-saturated with the insecticides (Table 2, Fig. 1A). It was suggested that the insecticides in the internal organic phase tended to move gradually to the external aqueous phase during the solvent evaporation process because they were slightly soluble in the polyvinyl alcohol-1788 (PVA-1788) solution. This would result in a decrease of the insecticide loading and the entrapment rate (Table 2). Moreover, the distribution of the insecticides within the microspheres would no longer be uniform due to the migration, which might further increase the insecticide release rate.

### Water addition in emulsion might encourage solvent evaporation and insecticide migration

Liu *et al*. and Zhang *et al*. investigated the effect of water addition in the emulsion on the morphology of spinosad-chlorpyrifos-microspheres^34, 35^. They found that microspheres with a smooth surface could be obtained if the emulsion was poured into 1,000 ml distilled water immediately after emulsification. In this study, the smooth-surfaced SP-EM-microspheres have been achieved with a similar treatment as well (Fig. S1). Moreover, the morphology of the microspheres did not vary significantly whether or not the external aqueous phase was saturated with the insecticides. Likewise, if 400 ml distilled water were added to the emulsion or PCL was applied together with PLA (at PLA:PCL = 5:1), or gelatin was used to coat the microspheres (at gelatin concentration = 2.5%) (Fig. S1). When distilled water was added to the emulsion, the concentration of emulsifier decreased. It might result in the decline of emulsifying ability, the size increase of emulsion droplet and the increase of the mean diameter of SP-EM-microspheres. On the other hand, the insecticide release rate increased with the increasing amount of water in the emulsion (Table 2, Fig. 1B). It was speculated that the increased contact area between air and water in the external aqueous phase encouraged solvent evaporation, which interfered with the encapsulation resulting in the higher than expected insecticide release rate^38, 39^. In the meantime, the migration of the insecticides from the internal organic phase to the external aqueous phase might be another key point.

### Add hydrophilic polymer to microsphere wall would promote insecticide release

Takayuki *et al*. attempted to increase the amount of the releasable Acetamiprid by incorporating PCL and PLA in the microspheres^25^. However, the molecular weight of PCL used in their study was lower than that of PLA. Suave *et al*. found that the release of Malathion loaded in the microspheres of pure poly (3-hydroxybutyrate) could be improved with the presence of PCL^26^. In this study, the SP-EM-microspheres prepared by using PCL as well as PLA of a similar molecular weight (ca. 80,000) confirmed the significant insecticide release increase (Fig. 1C) as well. Consequently, it was concluded that the added hydrophilic polymer for the microsphere wall would promote the insecticide release.

### Gelatin coating with microsphere would retard insecticide release

The release rate of neem oil (*Azadirachta indica* A. Juss) encapsulated with glutaraldehyde–alginate was reportedly significantly reduced or controlled by dipping the microspheres in a toluene solution of natural rubber^40^. Microspheres prepared with PLA and polyethylene glycol (PEG) were further coated with gelatin to exhibit a pronounced effect on preventing the initial burst of the drug release^41^. In this study, the gelatin-coated SP-EM-microspheres with a smooth surface also showed a significantly lowered insecticides release (Fig. 1D). It appeared that the gelatin coating could not only eliminate the initial surge and prolong the release of the insecticides, but also allow a degree of control on the microsphere’s properties for target applications.

### SP-EM-microspheres showed promising use for pest control as its long-term release effect

As compared to the naked SP and EM, the encapsulated insecticides displayed significantly higher photolysis stability provided by the protective effect of PLA against the sunlight degradation (Table 3, Fig. 2). This protection, along with the much-reduced release rate, afforded the SP-EM-microspheres a significantly improved long-term toxicity under sun exposure (Figs 1 and 2, Table 4). A similar phenomenon also existed in chlorpyrifos-microspheres using PLA for the wall material^36^. The surged toxicity of the SP-EM-microspheres at 48 h was slightly lower than that of the naked SP and EM. But the long-term toxicity of the SP-EM-microspheres against the larvae of *P. xylostella* under the field condition was excellent (Table 4) which could prove to be particularly desirable for aquatic pest control.

## Conclusion

**Figure 3:**
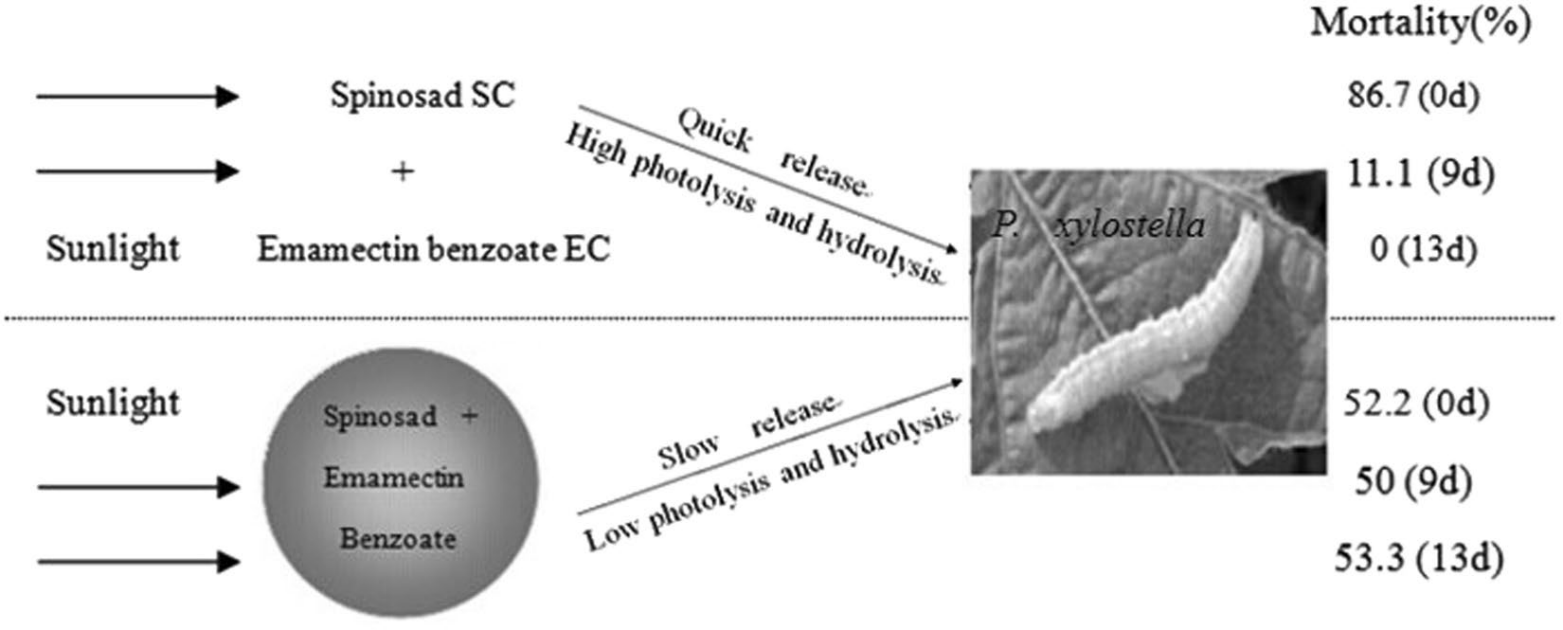
Graphical representation of research work. Biodegradable microspheres containing spinosad and emamectin benzoate with poly-lactic acid as wall material showed significantly lower release rate, photolysis and hydrolysis of the two insecticides, and higher long-term toxicity.

By using the solvent evaporation method, the biodegradable SP-EM-microspheres were prepared. The resultant microspheres offered significant control on the insecticide release, as well as a reduction on the photolysis and hydrolysis of the encapsulated insecticides. Various preparation conditions were studied to examine their effects on the microspheres. They included (1) saturation of the external aqueous phase with the insecticides, (2) addition of distilled water to the emulsion, (3) modification of the PLA/PCL ratio and (4) application of a gelatin coating. The physicochemical characteristics of the SP-EM-microspheres, such as the insecticide loading, entrapment rate, mean diameter, span and morphology, were slightly affected by the varied conditions. But, the release rate of the insecticides was found significantly reduced by the various preparation conditions of saturation of the external aqueous phase with the insecticides or application of a gelatin coating. On the contrary, the release rate could be accelerated by adding distilled water to the emulsion or incorporating hydrophilic polymer in the microsphere wall in preparing microspheres procedure. In comparison to the non-encapsulated SP and EM, the SP-EM-microspheres had a significantly higher long-term toxicity to *P. xylostella* due to the modified release as well as the reduced photolysis and hydrolysis of the insecticides (Fig. 3).

## Methods

### Materials

All of the experiments were conducted on the campus of Fujian Agriculture and Forestry University (FAFU) (34°480 N, 113°180 E), Fuzhou, Fujian, China. A field population of *P. xylostella* was collected from the commercial crucifer fields at Shangjie (Sj), 20 kilometers from FAFU. No specific permits were required for the collection, and no endangered or protected species were involved. The experimental protocol for the present study complies with the particular recommendation and has obtained necessary approval. The chemicals used were provided by the following manufacturers: Technical EM (95.8% pure emamectin benzoate) from Jiamusi Xingyu Biotechnique Development Co., Ltd., Heilongjiang, China; technical SP (84.9% pure spinosad), biodegradable PLA (technical grade, Mw = 80,000) and PCL (technical grade, Mw = 80,000) from Esun Industrial Co., Ltd., Shenzhen, China; PVA-1788 (chemically pure) from Aladdin Industrial Corporation; gelatin (chemically pure) from Sinopharm Chemical Reagent Co., Ltd., Beijing, China; Tube-O-Dialyzer (MWCO 300,000 Da) from Viskase Co., U.S.A.; Tesperse 2700 from Huntsman Co., U.S.A.; emulsifier polyoxyethylene (10) octylphenyl ether (OP-10) from Sinopharm Chemical Reagent Co., Ltd., Beijing, China; 2.5% SP SC from Dow AgroSciences, U.S.A.; and, 1.5% EM emulsifiable concentrate (EC) from Chambroad Holding Corporation, Shandong, China. Methanol and acetonitrile used for HPLC analysis were of chromatographical grade, and the others were analytical reagents.

### Preparation of SP-EM-microspheres

Based on our previous experiments to investigate the optimized ratio of SP:EM with higher toxicity to pest *P. xylostella* and the ratio of SP:EM recommended in other commercial formulations used in Chinese, the ratio of SP:EM = 4:1 was used to prepare microspheres containing SP and EM, the SP-EM- microspheres were prepared by the solvent evaporation method of Zhang *et al*. (2013). Briefly, 0.7336 g SP, 0.1625 g EM, and 0.7785 g PLA were mixed in 10 ml dichloromethane in an ice water bath. The mixture was, hereafter, in this study referred to as the “internal organic phase.” It was then emulsified in 100 ml previously prepared an aqueous solution of PVA-1788 (w/v = 2.0%) (hereafter, referred to as the “external aqueous phase”) at 3,600 r/min for 30 s using a high-speed disperser. The organic solvent in the emulsion was evaporated with a constant stirring at 560 rpm and 30 °C overnight. After 0.3 ml organic silicon defoamer was added, the microspheres gradually formed and were collected by centrifugation followed by air drying at 40 °C. Meanwhile, blank PLA microspheres containing no insecticides were also prepared following the same procedures.

### Effects of preparation conditions on insecticides release

The following microsphere preparation conditions were conducted to examine their effects on the insecticide release.

#### Saturation of external aqueous phase with insecticides

The external aqueous phase was saturated with SP and EM to prevent insecticide diffusion before the emulsion formation. At the same time, non-encapsulated, naked SP and EM, instead of SP-EM-microspheres, were used in the dialysis test for comparison.

#### Water addition in emulsion

The varied amount of distilled water (i.e., 0, 400 or 800 ml) was added to the emulsion to encourage solvent migration before evaporation during the formation of the microspheres.

#### Wall material variation

In addition to PLA, a synthetic polymer, PCL, was incorporated for the microsphere formation. The two polymers had a similar molecular weight. However, chemically, PCL was more hydrophilic than PLA.

#### Coating with gelatin

Approximately 0.5 g dried SP-EM-microspheres were dipped in 0.5, 2.5 or 4.5% gelatin aqueous solutions (25 ml) for 10 min at room temperature. They were centrifuged and dried in an oven at 40 °C for 24 h. SP-EM-microspheres coated with a thin gelatin film of varied thicknesses were thus obtained for the test on the insecticide release.

#### 5% SP-EM-microspheres suspension

Three grams of OP-10, 2.0 g Tesperse 2,700, and 5.0 g aluminum-magnesium silicate were dispersed in 77.7 g distilled water before 12.3 g SP-EM-microspheres were blended with ultrasound for 5 min. A 5% SP-EM-microspheres suspension (SP:EM = 4:1) was thus obtained.

### Characteristics of SP-EM-microspheres

#### Insecticide loading and entrapment rate

The insecticide loading was the actual contents of SP and EM in the microspheres. The entrapment rate was the ratio of actual-to-theoretical contents of the particular insecticide. In 2 ml dichloromethane, 0.02 g SP-EM-microspheres was dissolved. After the dichloromethane had evaporated completely, the residue was washed with ultrasound five times with 1.5 ml methanol each time. The methanol solutions were collected and diluted to 10 ml for the determination of SP and EM concentrations by HPLC. The entrapment rate was also calculated.

#### Mean microsphere diameter and span

To determine the diameter of the microspheres, 0.3 g SP-EM-microspheres and 0.3 g sodium phosphate were ultrasonically mixed in 40 ml distilled water. It was measured using LS-POP (7) Laser particle size analyzer (OMEC Instrument Co., Ltd., Zhuhai, China) after rapid sample introduction. The microsphere span was calculated as (*D*_90_−*D*_10_)/*D*_50_. Where, *D*_50_ was the mean diameter of the microspheres, *D*_90_, the diameter of 90% of the microspheres, and *D*_10,_ that of 10% of the microspheres.

#### Morphology of SP-EM-microspheres

Morphology of the SP-EM-microspheres was observed under a light microscope from time to time during preparation. Dry SP-EM-microsphere samples were sputter-coated with gold by JFC-1200 Sputter Coater 9 (Japan Electron) for scanning by a scanning electron microscope (JSM-5310LV, Japan Electron).

### Determination of SP and EM content

#### HPLC

Contents of SP and EM were determined by HPLC with UV detection by using an Amemyst c18-H column at 25 °C. The mobile phase composed of a 0.5% methanol-acetonitrile-sodium acetate solution (45:45:10) with a flow rate of 1.0 ml/min. The detection wavelength was 245 nm, and the injection amount was 20 μl. The insecticide content was calculated from the regression equation established between the known insecticide concentrations and their corresponding HPLC peak areas.

#### Spectrophotometry

Phosphate buffer solutions (pH 7.0 containing 20% methanol) of SP and EM at the concentrations of 0.005, 0.01, 0.02, 0.03, 0.04, and 0.05 mg/ml (with a SP:EM ratio of 4:1) were prepared. The same buffer solution without insecticides was used as control check (CK). Optical density (OD) of a 2.0 ml solution in a quartz cup on a spectrophotometer was obtained at 250 nm. SP or EM content was calculated from the regression equation established with known insecticide concentrations and their corresponding ODs.

#### Insecticides release of SP-EM-microspheres

The barrier diffusion method^42^ was applied to study the insecticide release of the SP-EM-microspheres using a phosphate buffer solution (pH 7.0) containing 20% methanol as the release medium. Briefly, 150 mg (for HPLC) or 40 mg (for spectrophotometer) SP-EM-microspheres were accurately weighed and placed in a dialysis tube (Tube-O-Dilalyzer, MWCO 300,000 Da) filled with 2.0 ml release medium. Then, the two ends of the dialysis tube were tightly tied and placed in a conical flask containing 150 ml release medium with constant shaking at the speed of 120 r/min at 25 °C in the dark. Two ml (for HPLC) or 4.0 ml (for spectrophotometer) of the release medium were pipetted from the flask for sampling after 2, 4, 7, 10, 13, 15, 15 and 24 d. The concentrations of the released insecticides were determined. At the time of sampling, 2.0 ml (for HPLC) or 4.0 ml (for spectrophotometer) of freshly prepared release medium was added to replenish the pool in the flask.

#### Insecticides release of SP, EM, and SP + EM

Using HPLC, the release rates of SP, EM, and SP + EM could be detected separately, while the spectrophotometer could only provide the combined release of SP + EM. The accumulated release rate of an insecticide or insecticide combination was defined by dividing the insecticide (or insecticide combination) released in the release medium during a given period by its original concentration at zero time. Based on the data collected at different sampling time (d), a release curve and a Peppas equation^23^, i.e., (M_t_/M_z_ = kt^n^), for each insecticide or combination of insecticides were established. *T*_50_ was calculated according to the Peppas equation. At least three replicates were performed for each assay.

#### Photolysis of SP-EM-microspheres

At 0.04 g, dry SP-EM-microspheres or a mixture of blank PLA microspheres (without the insecticides) with naked SP and EM were placed in a small glass beaker, sealed and kept under natural light. The degradation rates of SP and EM in the samples were determined by HPLC after 0, 2, 4, 6, 10 and 15 d. The insecticide degradation rate was calculated as the ratio of the concentration of SP or EM at a given time vs. that at zero time. A kinetic equation was established from the degradation curve. More than three replicates were performed for each assay.

### Photolysis and hydrolysis on insecticides exposed to sunlight

#### LC_50_ bioassay

Using a leaf dipping method^43, 44^, the toxicity of SP-EM-microspheres suspension against 3^rd^ -instar larvae of *P. xylostella* was determined at 25 °C with a photoperiod of 16:8 (light:dark) in the laboratory. According to previous tests, the concentrations at which the corrected death rate ranged from 10% to 90% were selected as the treatment concentrations. 5 concentrations of the mixture of 2.5% SP suspension concentrate and 1.5% EM emulsifiable concentrate (maintaining an SP:EM = 4:1) were used to calculated LC_50_ values (0.025, 0.050, 0.300, 0.630, 1.260 mg/L). And 6 concentrations of SP-EM-microspheres suspension were used to determine LC_50_ values (0.075, 0.150, 0.300, 0.630, 1.260, 2.520 mg/L). Discs of cabbage (*Brassica oleracea* L.) leaves, 5 cm in diameter, were dipped in these pre-prepared suspensions for 15 s and then taken out and air dried naturally. Subsequently, the leaves were placed into petri dishes laid with wet filter paper. Afterward, new 3^rd^ -instar larvae of *P. xylostella* were put into the petri dishes (10 larvae per replication). Three biological replicates and control were used for each microsphere concentration. Mortalities of the *P. xylostella* larvae after 48 h were recorded. If the larvae had no autonomous response when they were touched with a writing brush, they were proved to be dead. Zero mortality was observed in distilled water as a control. No specific permissions were required for our collection of diamondback moths because the scientists were welcome to collect the insect samples from the farmers’ crucifer fields to control the pest insects. The field studies did not involve endangered or protected species.

#### Toxicity of SP-EM-microspheres suspension after sun degradation

Two preparations of MS and MX was made by adding distilled water to arrive at a same final concentration of 1.2 mg/L (i.e., SP = 0.96 mg and EM = 0.24 mg with an M:M ratio of 4:1). Then, 100 ml each of the preparation were kept in a beaker covered with a plastic film under sunlight. Toxicity of the preparations was determined on 0, 2, 7, 9, 13, 17 and 21 d afterward, which were operated in the conditions of aforementioned LC_50_ bioassay method. 30 larvae of *P. xylostella* per replication, three biological replications and control were tested in each toxicity experiment. Meanwhile, the preparations for testing were diluted with distilled water to 100 ml.

## Electronic supplementary material

Supplementary Information
