# Supplementary material for: Release and Degradation of Microencapsulated Spinosad and Emamectin Benzoate
Source: Sci Rep. 2017 Sep 7;7:10864. doi: 10.1038/s41598-017-11419-2 (PMC5589813; doi:10.1038/s41598-017-11419-2)
Supplement: Supplementary file 1 — Supplementary Information [file 41598_2017_11419_MOESM1_ESM.doc]

**Release and Degradation of Microencapsulated Spinosad and Emamectin Benzoate**

**Bin Bin Huang^1, 2^, Shao Fei Zhang^1, 2^, Peng Hao Chen^1^, Gang Wu^1, 3^**

^1^ Key Laboratory of Biopesticide and Chemical Biology (Ministry of Education), Fujian Agriculture and Forestry University, Fuzhou, 350002, China

^2^ These authors contributed equally to this article

^3^ Corresponding author: Gang Wu, e-mail: newugan@163.com

**Supplementary Information**

**Supplementary materials and methods**

In our previous study, the polylactic acid microspheres containing spinosad and emamectin benzoate were prepared by using solvent evaporation method, influence of content of polylactic acid in oil phase, theoretical pesticide loading and stirring speed in the preparation process was investigated. The actual pesticide loading, entrapment rate, mean diameter, span and release percentage in 5 days were studied. In main text of this manuscript, releasing and degradation characteristics of microspheres were investigated. In addition, some factors which influenced the physicochemical characteristics of SP-EM-microspheres in the preparation were showed as follows.

**Surfactant types**: In this study, gum arabic, gelatin, PVA-1788 were used as surfactant in the same content (1.5%, w/w) respectively. And influence of different surfactant on characteristics of microspheres was studied.

**Content of surfactant**: The content of surfactant was a decision factor which influenced the surface tension of emulsion. In our study, influence of different content (1.0%, 1.25%, 1.5%, 1.75%, 2.0% w/v) of PVA-1788 on characteristics of microspheres was studied.

**Volume ratio of organic phase to aqueous phase:** In our study, influence of different volume ratio of internal organic phase (mixture of methylene chloride, PLA, spinosad and emamectin benzoate) to external aqueous phase (PVA-1788 solution, 1:10, 1:15, 1: 20, 1: 25) on characteristics of microspheres was studied.

**Supplementary results**

**Surfactant types:** When gum arabic or gelatin was used as surfactant, emamectin benzoate microspheres could be obtained successfully, but entrapment rate of microspheres compared using PVA-1788 was higher than the entrapment rate of microspheres compared using gum arabic or gelatin. In addition, mean diameter of microspheres prepared from PVA-1788 was smaller comparing with microspheres prepared from gum arabic or gelatin. So, PVA-1788 was selected as surfactant.

**Content of surfactant:** When the content of PVA-1788 varied from 1.0% to 2.0%, the entrapment rate and diameter of microspheres varied from 49.45±1.45% to 67.32±3.48% and 14.63±0.52μm to 12.45 ±0.45μm and particle size distribution of microspheres decreased (Table S2). So 2.0% content of PVA-1788 was selected.

**Volume ratio of organic phase to aqueous phase:** When Volume ratio of organic phase to aqueous phase varied from1:5 to 1:25, the mean diameter of microspheres varied from 8.29 ±0.203μm to 13.71 ±0.53μm gradually (Table S3). When the volume ratio of organic phase to aqueous phase was 1:10, the entrapment rate of microspheres (=69.32±1.84) was highest significantly (Table S3). So 1:10 was selected as the volume ratio of organic phase to aqueous phase.

**Table S1. Effects of surfactant on the preparation of microspheres**

| Type | Pesticides loading (%) | Entrapment rate (%) | Mean diameter D_50_(μm) | Span |
| --- | --- | --- | --- | --- |
| gum arabic | 6.83±0.55 | 35.19±2.81 | 15.75±0.69 | 1.315±0.064 |
| gelatin | 8.69±0.36 | 44.78±1.87 | 13.32±0.29 | 1.309±0.019 |
| PVA-1788 | 11.64±0.31 | 60.00±1.60 | 12.78±0.78 | 1.366±0.106 |

**Table S2. Effects of Content of PVA-1788 on the preparation of microspheres**

| Content of PVA-1788(%) | Pesticide loading (%) | Entrapment rate (%) | Mean diameter  (μm) | Span |
| --- | --- | --- | --- | --- |
| 1.00 | 9.60±0.28 | 49.45±1.45 | 14.63±0.52 | 1.407 ±0.045 |
| 1.25 | 10.67±0.20 | 54.95±1.04 | 13.13±0.92 | 1.236 ±0.098 |
| 1.50 | 11.65±0.36 | 60.00±1.87 | 12.79 ±0.78 | 1.366±0.106 |
| 1.75 | 11.88±0.28 | 61.18±1.42 | 12.64 ±0.90 | 1.210 ±0.017 |
| 2.00 | 13.07±0.68 | 67.32±3.48 | 12.45 ±0.45 | 1.298 ±0.012 |

**Table S3. Effects of Volume ratio of organic phase to aqueous phase on the preparation of microspheres**

| Volume ratio of organic phase to aqueous phase | Pesticide loading (%) | Entrapment rate (%) | Mean diameter  (μm) | Span |
| --- | --- | --- | --- | --- |
| 1: 5 | 12.84±0.26 | 66.16±1.33 | 8.29 ±0.203 | 1.307 ±0.033 |
| 1:10 | 13.46±0.36 | 69.32±1.84 | 10.41 ±0.49 | 1.374 ±0.012 |
| 1:15 | 12.29±0.38 | 63.31±1.93 | 11.09±025 | 1.344 ±0.113 |
| 1:20 | 11.65±0.35 | 60.00±1.87 | 12.65 ±0.56 | 1.366 ±0.106 |
| 1:25 | 12.91±0.24 | 66.51±1.36 | 13.71 ±0.53 | 1.359±0.021 |

| 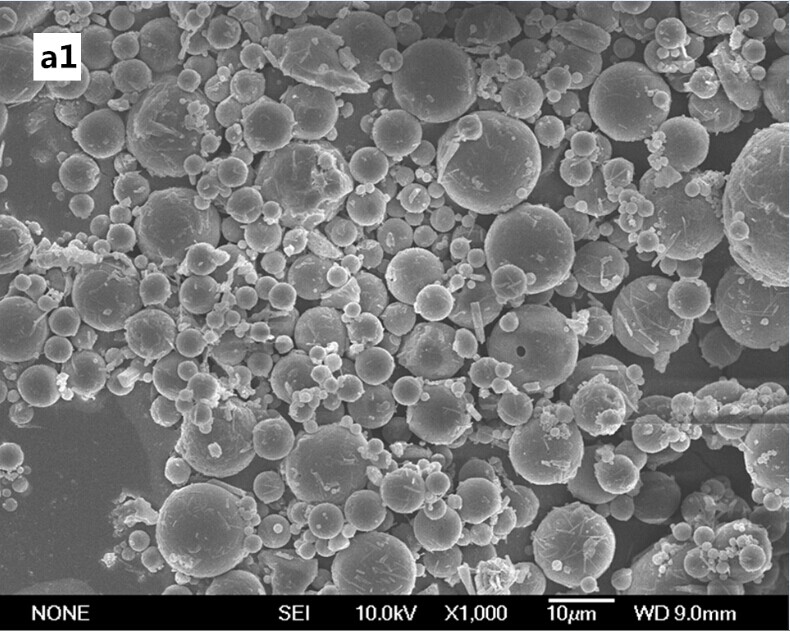 | 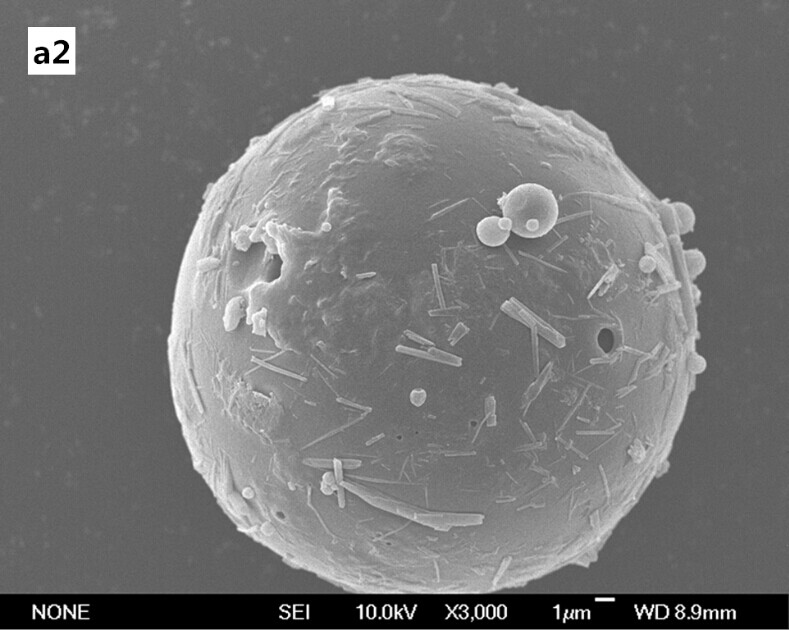 |
| --- | --- |
| 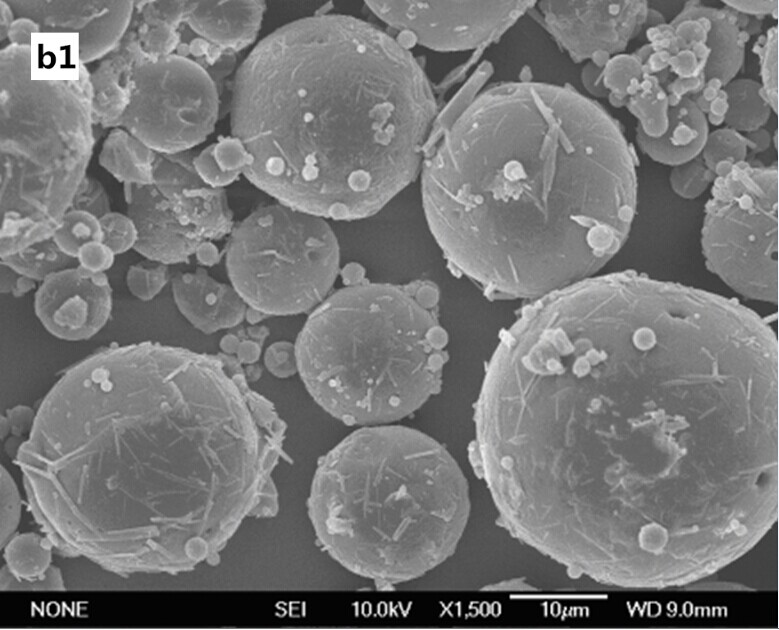 | 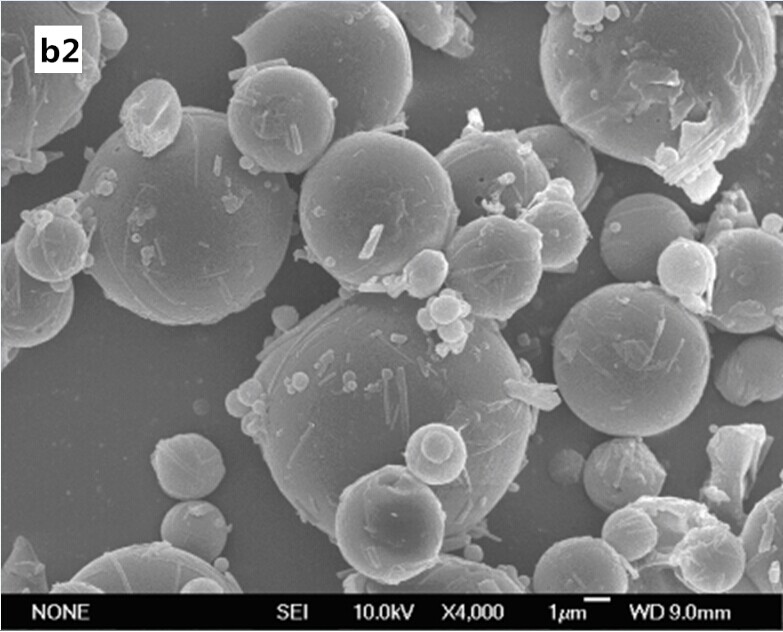 |
| 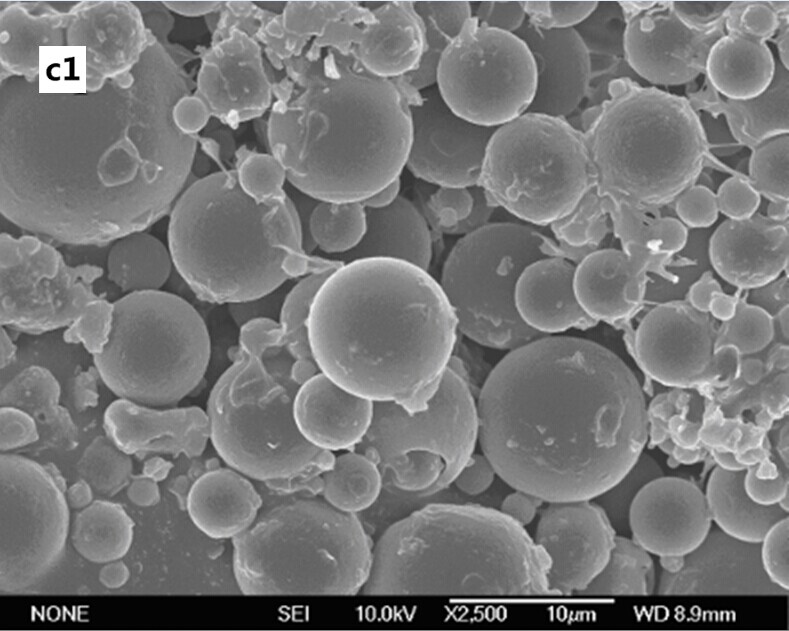 | 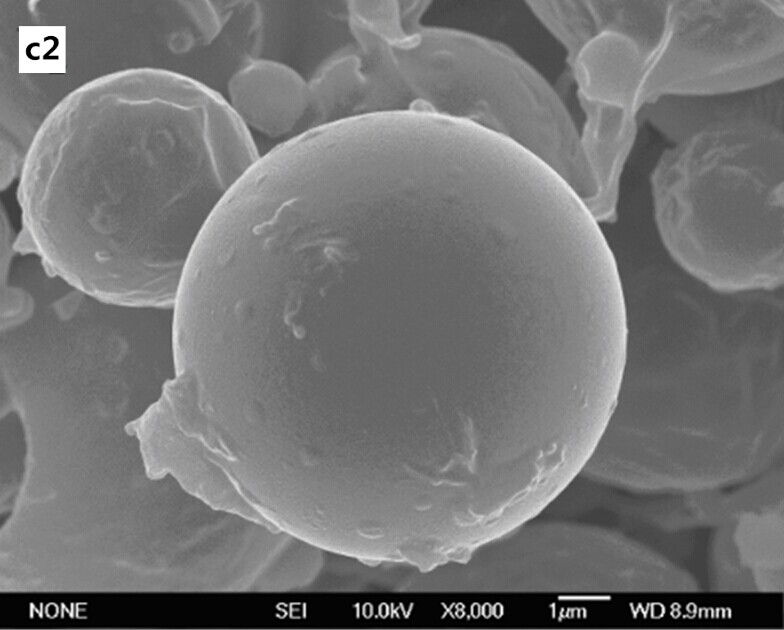 |
| 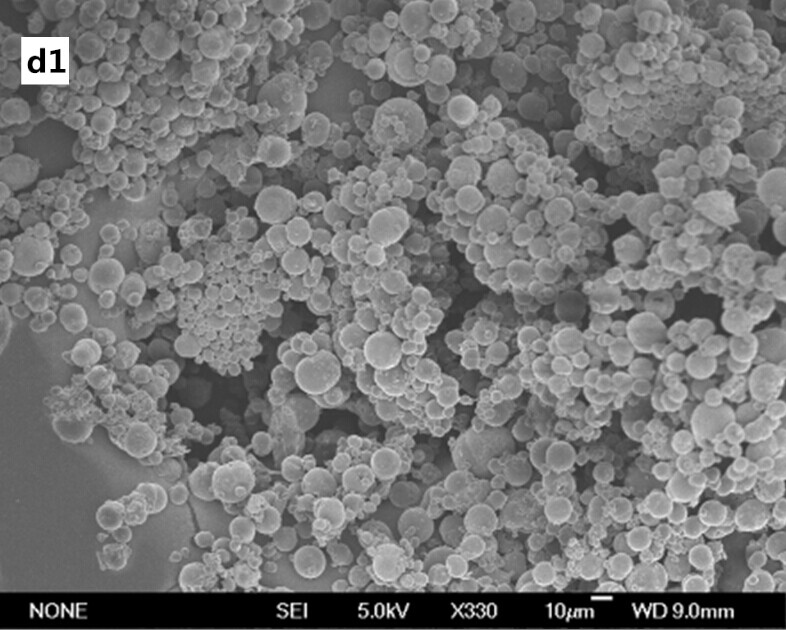 | 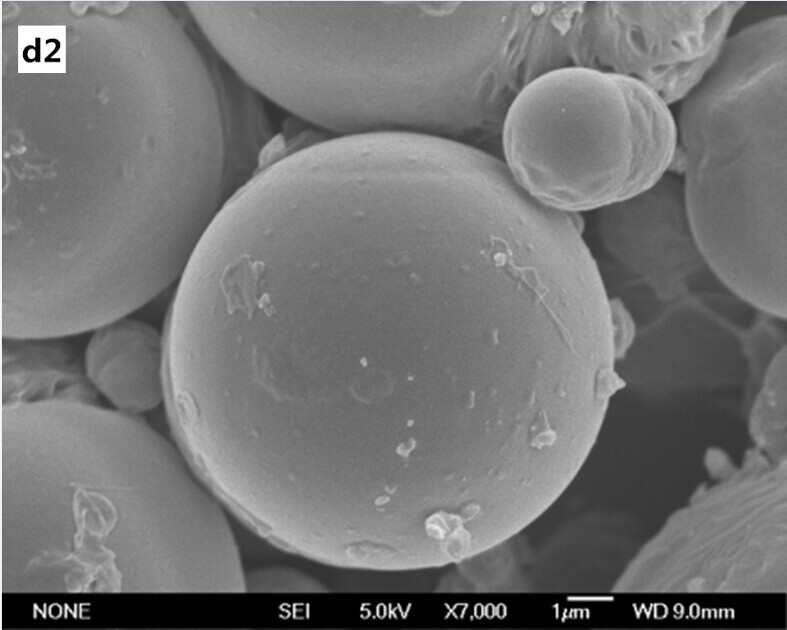 |
| 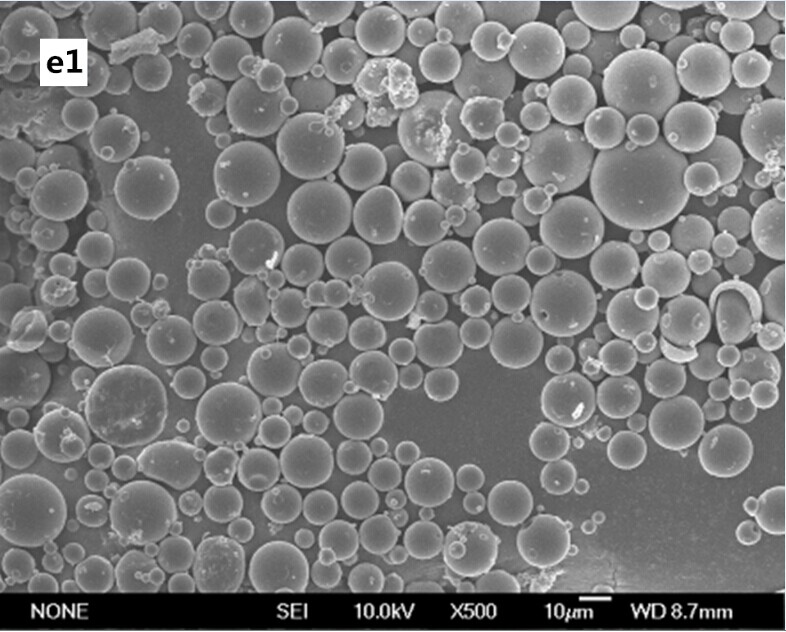 | 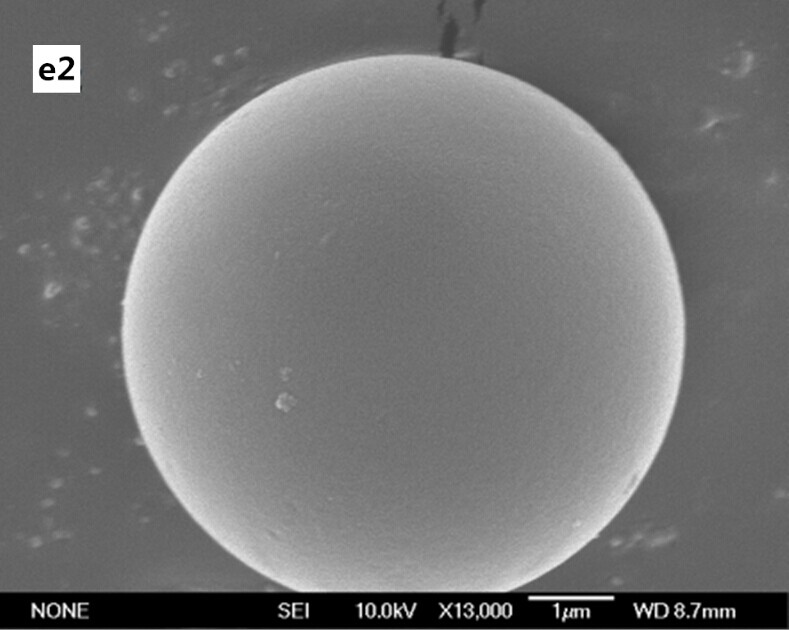 |
| **Figure S1. Scanning electron microscope photographs of SP-EM-microspheres.** a1 and a2 represents microspheres prepared by the typical solvent evaporation method, i.e. external aqueous phase was not saturated by adding spinosad and emamectin benzoate into external aqueous phase, the emulsion solution was stirred directly without adding distilled water, PLA only was used as wall materials, and no gelatin was used as coating gelatin film of microspheres. b1 and b2 represents microspheres were prepared when external aqueous phase was saturated with spinosad and emamectin benzoate; c1 and c2 represents microspheres were prepared by using the modified solvent evaporation method (i.e., adding distilled water into emulsion solution, and the volume of distilled water=400 ml); d1 and d2 represents microspheres were prepared when PLA and PCL were used as wall materials at a ratio of PLA/PCL=5:1; e1 and e2 represents microspheres were prepared when gelatin (content of gelatin=2.5%) was used as coating gelatin film. | |
